# Portable Biosensors for Psychophysiological Stress Monitoring of a Helicopter Crew

**DOI:** 10.3390/s20236849

**Published:** 2020-11-30

**Authors:** Marta Vicente-Rodríguez, Damián Iglesias Gallego, Juan Pedro Fuentes-García, Vicente Javier Clemente-Suárez

**Affiliations:** 1 Psychophysiological Research Group, European University of Madrid, Tajo Street, s/n, Villaviciosa de Odón, 28670 Madrid, Spain; Martavr94@gmail.com; 2 Physical Education & Exercise Lab, Teacher Training College, University of Extremadura, 10003 Cáceres, Spain; diglesia@unex.es; 3 Faculty of Sport Science, University of Extremadura, Avda. Universidad S/N, 10003 Cáceres, Spain; 4 Faculty of Sport Sciences, Universidad Europea de Madrid, Villaviciosa de Odón, 28670 Madrid, Spain; vctxente@yahoo.es; 5 Grupo de Investigación en Cultura, Educación y Sociedad, Universidad de la Costa, Barranquilla 080020, Colombia

**Keywords:** stress, experience, military, heart rate variability, anxiety

## Abstract

This study aims to analyze the psychophysiological stress response of a helicopter crew using portable biosensors, and to analyze the psychophysiological stress response differences of experienced and non-experienced crew members. We analyzed 27 participants (33.89 ± 5.93 years) divided into two different flight maneuvers: a crane rescue maneuver: 15 participants (three control and 12 military) and a low-altitude maneuver: 12 participants (five control and seven military). Anxiety, rating of perceived exertion, subjective perception of stress, heart rate, blood oxygen saturation, skin temperature, blood lactate, cortical arousal, autonomic modulation, leg and hand strength, leg flexibility, spirometry, urine, and short-term memory were analyzed before and after both helicopter flight maneuvers. The maneuvers produced a significant increase in stress and effort perception, state of anxiety, and sympathetic modulation, as well as a significant decrease in heart rate, blood oxygen saturation, leg and inspiratory muscle strength, and urine proteins. The use of biosensors showed how a crane rescue and low-altitude helicopter maneuvers produced an anticipatory anxiety response, showing an increased sympathetic autonomic modulation prior to the maneuvers, which was maintained during the maneuvers in both experienced and non-experienced participants. The crane rescue maneuver produced a higher maximal heart rate and decreased pulmonary capacity and strength than the low-altitude maneuver. The psychophysiological stress response was higher in the experienced than in non-experienced participants, but both presented an anticipatory stress response before the maneuver.

## 1. Introduction

Previous studies in the military population have shown how combat is one of the most extreme experiences that a person can face [1]. Independently of the combat situation, in military deployments, there are unpredictable and unknown hazards that elicit a stress response in the organism [2]. This response activates the fight–flight system and the physiological mechanisms that are basic to survival, also affecting cognitive processes, such as information processing, memory, perception, attention, judgement, and decision-making, as well as physical aspects, such as endurance, strength, and flexibility [1,3,4,5,6]. The continuous exposure to the combat stressors of military populations and air force crews have been previously related with mental disorders, such as post-traumatic stress disorder (PTSD) [7], highlighting the importance of personalized training based on a better understanding of the psychological and physiological responses involved in stress coping mechanisms.

Military air operations are considered some of the most stressful events in the military, but most previous aircrew research in this area has been carried out on special tactical groups, such as paratroopers, showing how the exposure to these extreme environments and types of jumps produced a decrease in cortical arousal and an increase in the physiological stress response, mediated by the autonomous sympathetic system [8,9,10,11]. In particular, aircrews are susceptible to suffering a large variety of critical situations, since they are exposed to stressors such as hypoxia, disorientation, G forces, accidents, or air attacks during their interventions, and have to face different demands, such as different types of parachute jumps, nocturnal jumps, ground training in war zones, and different types of flights at different heights—HALO (high altitude low opening) and HAHO (high altitude high opening)—as well as underwater training in case of aircraft accidents between others [6,7,8,9,10,11,12,13,14,15,16,17,18].

In this line, some air forces’ special units, such as helicopter search and rescue (SAR) crews, have to conduct different flying maneuvers to reach their objectives; these highlight low-altitude flights where attention, focus, and contextual information processing are the basis, as well as crane rescues of wounded, where a rescuer descends by crane to carry out the rescue while the rest of the crew stabilize the helicopter in height. Both crane rescue and low-altitude maneuvers present extra stress and danger for aircrews, as they are some of the most dangerous and demanding of this type. The psychophysiological demands of these two maneuvers have not yet been defined, despite the importance of designing specific training for these dangerous and demanding situations. As stress is a major concern in this field, the analysis of stress reactions in such extreme environments has a wide range of benefits in terms of learning and improving actual knowledge, but it also requires instruments that are easy to use, durable, portable, noninvasive, and validated; otherwise, it could be difficult to measure physiological stress markers during maneuvers in real time. A salivary amylase biosensor has recently been used for assessing stress, but it has not been deeply examined in the military combat environment [19]; there are also other wearable noninvasive chemical sensors for detecting chemical biomarkers from tears, sweat, and saliva. Saliva is the one with good correlation with blood concentrations of analytes such as lactate, a substance related with fatigue and physical exertion, but the actual evidence is poor in this field [20]. For this reason, we proposed the present research focused on the analysis of two demanding maneuvers: a rescue crane maneuver and low-altitude flight. The first objective of this research was to analyze the psychophysiological stress response of helicopter crews using portable sensors monitoring vital signs in two real flight maneuvers: a crane rescue maneuver and a low-altitude flight maneuver. In addition, the second objective was to analyze the differences in the psychophysiological responses of experienced and non-experienced crew members due to the special relevance of experience as a protective factor. We hypothesized that (i) a rescue crane maneuver would elicit a higher psychophysiological stress response than a low-altitude flight due to the higher danger of the operation and the extra stress of control of the wounded during the maneuver, and (ii) non-experienced personnel would present a higher psychophysiological stress response than experienced personnel in helicopter maneuvers.

## 2. Materials and Methods

### 2.1. Participants

We analyzed a total of 27 participants (Table 1); 19 were military personnel with training and experience, and 8 were civil participants without any type of military experience or knowledge in the fields of helicopters or rescue, which served as a control group. The participants were divided randomly by the military chief into two groups depending on the flight: (1) low-altitude flight with 12 subjects in total, 5 control and 7 military (33.91 ± 5.53 years; 172.08 ± 4.48 cm; 70.08 ± 9.70 kg; 9.08 ± 8.70 years of service; 2.91 ± 4.54 months in mission; 401.67 ± 510.16 flying hours); and (2) crane rescue maneuver with 15 subjects, 3 control and 12 military (33.86 ± 6.42 years; 178.01 ± 8.56 cm; 76.82 ± 14.46 kg; 12.65 ± 9.45 years of service; 5.73 ± 4.96 months in mission; 767.16 ± 704.74 flying hours).

### 2.2. Procedure

Before participation, the experimental procedures were explained to all the participants, who gave their voluntary written informed consent following the Declaration of Helsinki. All the procedures were approved by the Head Quarter of the Unit, the Military Ethics Committee (68/18), and the University Ethics Committee (CIPI/18/093). The entire research procedure was conducted with standard flying suits, boots, harnesses, helmets, and earphones, corresponding with the weight of the operating equipment for this type of action.

The study was conducted over a week, with 3 days between the two maneuvers. The procedure of collecting psychological variables involved in both maneuvers was carried out first using paper and pencil. The maneuvers were divided into three moments: pre-, during-, and post-intervention. In the pre- and post-moments, the researchers collected the samples in the same conditions in the SAR unit hangars.

### 2.3. Research Design, Instrumentation, and Study Variables

An experimental and prospective pre–post intervention was conducted to analyze the following psychological and physiological variables before and immediately after the helicopter maneuvers:

- Rating of perceived exertion (RPE), Borg 6–20 scale [21].
- Subjective stress perception (SSP) on a 1–100 scale [22].
- Flexibility in the sit-and-reach test using a sit-and-reach box. This system allows one to evaluate subjects’ hamstring flexibility, and the sliding ruler centered above the box was used to obtain the flexibility scores before and after the maneuver [14]. The measurement of this variable was obtained before the performance of the horizontal jump to analyze their basal flexibility.
- Blood oxygen saturation (SatO2) and heart rate (HR), measured using a finger pulse-oximeter system (PO 30 Beurer Medical, Ulm, Germany).
- Blood lactate concentration, measured using the Lactate Pro II Arkay, Inc. system (Kyoto, Japan) [14].
- Skin temperature (ST°), measured using a digital infrared thermometer (Temp Touch; Xilas Medical, San Antonio, TX, USA).
- Cortical arousal and fatigue of the central nervous system (CNS), measured using the average of 5 incremental tests (20 to 100 Hz) with the Lafayette Instrument critical flicker fusion threshold (CFFT) (Model 12021), according to previous authors [6]. For the database, we collected all the attempts and used the mean of the five results for the statistical analysis.
- Lower-body muscular strength manifestation, measured using a horizontal jump test. Subjects performed a standardized warm-up consisting of 2 × 10 vertical jumps, and then they performed two maximal horizontal jumps, as a previous report instructed, and the best attempt was used for the statistical analysis [23].
- Isometric hand strength (IHS), measured using the grip dynamometer TKK 5402 (Takei Kiki Koyo, Japan). Participants had two attempts before and after the maneuver, and the best result was used in the data.
- Strength of the respiratory muscles, measured using the spirometry variables of forced vital capacity (FVC), volume exhale at the end of the first second of forced expiration (FEV1), and the peak expiratory flow (PEF) using a QM-SP100 (Quirumed, Valencia, Spain) spirometer in a maximum inhale–exhale cycle.
- Hydration, measured using the urine color chart [18].
- Urine protein, glucose, nitrates, and pH, measured using the Urine Combur-Test (Roche, Madrid, Spain) stripes.
- Urine specific gravity (USG), measured using a handheld digital refractometer (Atago PAL-10S, Bellevue, WA, USA).
- Cognitive anxiety (CA), somatic anxiety (SA), and self-confidence (SC), measured using the Revised. Competitive State Anxiety Inventory-2 (CSAI-2R) questionnaire, as well as the anxiety state, measured using the state-trait anxiety questionnaire (STAI) [14,24].
- Short-term memory (S-TM), measured using a three-digit number showed to the participant for 1 s, and after 5 s, they were asked to say the numbers in the direction opposite to the one initially exposed.

The autonomic modulation of participants was evaluated using the heart rate variability (HRV) [25]. We used the Polar V800 HR monitor (POLAR, Finland) to record the HRV data and the Kubios HRV software 3.1.0 (Kuopio, Finland) to analyze the HRV data [26]. We divided the samples into three moments: pre-flights (20 min before the flight), the flight (130 min), and post-flight (20 min after finishing the flight). For the pre-flights and post-flights, participants were seated in a quiet and temperature-controlled room (25 °C). During the flights, participants were in their tactical positions doing their normal tasks in order to conduct the most efficient research. The whole intervention was observed by control subjects, and in the pre- and post-moments, the Polar V800 HR monitor was placed, started, and stopped by control participants.

We analyzed the variables of the mean HR, minimum HR (Min HR), maximum HR (Max HR), the square root of the mean value of all sums of square differences of all R-Rs (temporal differences between R waves of electrocardiogram) that followed intervals (RMSSD), and the percentage of differences between normal adjacent R-R intervals greater than 50 ms (pNN50) of the temporal domain. In addition, we analyzed the low-frequency band (LF), the high-frequency band (HF), the ratio of low frequency to high frequency (LF/HF Ratio) of the frequency domain, the sensitivity of the short-term variability (SD1) of the nonlinear domain, and the sensitivity of the long-term variability (SD2) of the nonlinear domain. For the analysis, we used very low artifact correction, a detrending method that removed very low-frequency trend (frequencies below 0.04 Hz), and an autoregressive (AR) spectral analysis method.

### 2.4. Flying Maneuver

The helicopter’s flying maneuvers were performed in an AS-332 Super Puma helicopter. The evaluations were divided into three phases:

1. Pre-maneuver (20 min before the flight maneuver), where basal variables were taken.
2. Maneuver (130 min in which the flight maneuver was conducted).
3. Post-maneuver (20 min after the flight maneuver), where post-evaluations were made.

**Figure 1:**
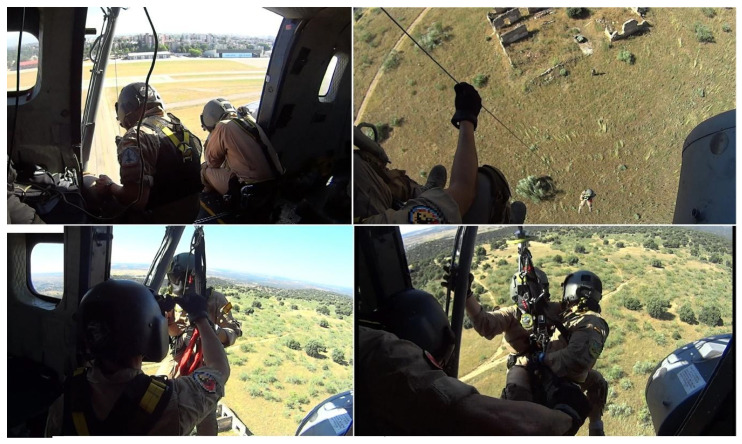
Different moments of the crane rescue maneuver.

**Figure 2:**
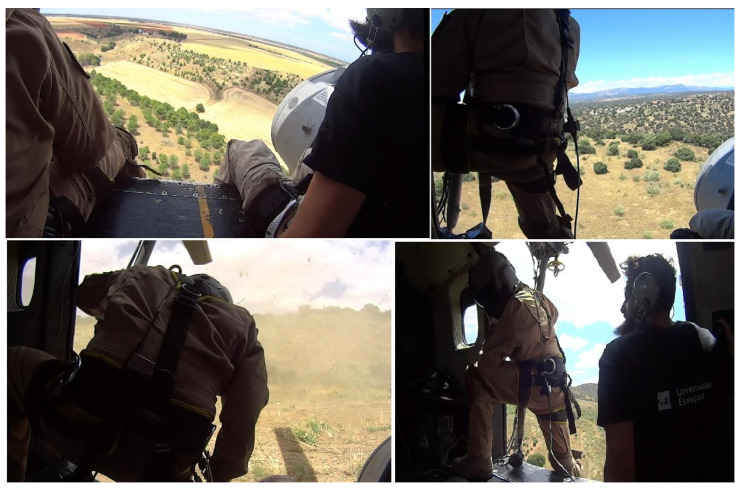
Different moments of the low-altitude flight maneuver.

In the rescue crane maneuver, the helicopter made an approximation to an elevated zone where the pilots stopped the helicopter in flight and the crew got out of the helicopter and carried out an incursion in a simulated war zone in which they recovered a wounded soldier and rescued them using the crane (Figure 1). For the crane rescue, one participant was waiting inside the helicopter, near the open door, wearing a harness and controlling the crane controls, while the rescuer took care of the wounded during the elevation process. The low-altitude flight maneuver consisted of three approximations to different military points, flying at low altitude over valleys and mountainous areas (Figure 2).

### 2.5. Statistical Analysis

The SPSS statistical package (version 24.0; SPSS, Inc. Chicago, IL, USA) was used to analyze the data. Normality and homoscedasticity assumptions were checked with a Shapiro–Wilk test, showing a no parametric distribution. To reach the first objective, we analyzed differences between pre- and post-samples using the Wilcoxon and Friedman tests. To reach the second objective, differences between groups were analyzed using the Mann–Whitney and Kruskall–Wallis tests. Bonferroni correction was applied to analyze the data. The effect size (ES) was tested by Cohen’s D (ES = (Post-test mean-Pre-test mean)/Pre-test SD). The level of significance for all the comparisons was set at *p* ≤ 0.05.

## 3. Results

The results are reported as medians and in parenthesis (percentile 25–percentile 75). There were no significant differences between military personnel in the crane rescue and low-altitude maneuvers in years of service, months in mission, or flying hours. As the control group did not have any military experience, the personnel of the crane rescue and low-altitude maneuvers presented significantly higher years of service, months in mission, and flying hours.

The SSP and RPE significantly increased after the crane rescue and low-altitude flights, and were higher in the crane rescue maneuver than in the low-altitude flight. Skin temperature showed a significant decrease in the post-values of the crane rescue maneuver in comparison with the pre-values. The HR variables showed a significant decrease in the post-values of the low-altitude maneuver in comparison with the pre-values. The HR was higher in the pre-values of the crane rescue maneuver than in the low-altitude flight. SatO2 showed a significant decrease in the post-values of the rescue crane maneuver than in the pre-values, and showed significant differences between both flights; it was higher in the pre-values of the crane rescue maneuver compared with the low-altitude flight. The Horizontal jump, FEV1, and PEF showed a significant decrease after the crane rescue maneuver. Urine proteins showed a significant decrease after both flights. STAI-S showed a significant increase after the crane rescue maneuver (Table 2).

The pre/post results of the psychophysiological variables in the military and control participants are shown in Table 3. The SSP, RPE, and HR showed a significant increase, and the FEV1, PEF, and urine protein showed a significant decrease after the flights for the military participants. The CFFT showed a significant decrease in the control participants after the flights.

The pre-, during, and post-results of the HRV variables in the crane rescue and low-altitude flights are shown in Table 4. The mean HR and Max HR were significantly higher in the crane rescue maneuver than in the low-altitude flight. LF showed a significant decrease during and after the low-altitude flight, and was lower in the post-values, presenting the opposite result for LF. The LF/HF ratio showed a significant decrease during and after the low-altitude flight, and was lower in the post-values. SD2 showed a significant decrease during the low-altitude flight and an increase in the post-values.

## 4. Discussion

The aims of this study were, first, to analyze the psychophysiological stress response of a helicopter crew using portable sensors in real flight maneuvers—a crane rescue maneuver and a low-altitude flight—and second, to analyze the differences in the psychophysiological responses of experienced and non-experienced crew members. The hypothesis (i), which claimed that a rescue crane maneuver would elicit a higher psychophysiological stress response than a low-altitude flight due to the higher danger of the operation and the extra stress of control of the wounded during the maneuver, was confirmed, since the psychophysiological stress response in the crane rescue maneuver was higher than that in the low-altitude flight maneuver. The hypothesis (ii), which claimed that non-experienced participants would present a higher psychophysiological stress response than the experienced personnel in helicopter maneuvers, was partially confirmed, since the psychophysiological stress response was higher in the military personnel than in control participants, but both presented an anticipatory stress response before the maneuver.

We found an increase in subjective stress perception after both flights that was significantly higher in the crane rescue than in the low-altitude flight maneuver, probably due to the characteristics of the crane rescue maneuvers, in which participants should rescue a wounded person while the helicopter is controlled by the pilot, copilot, and mechanic, and the wounded person must be stabilized, secured, and brought aboard the helicopter by the rest of the crew. This response was contrary to the previously reported habituation to stressors, highlighting the difficulty and demands of the maneuver and the consequent increased stress response [9]. This response could be related to the lack of control of the environment and the constant and relevant information they must process to successfully complete the task of rescuing, which maintain the stress activation [9]. The large demands of both flights were reflected in the large lactate values after both maneuvers, which were above the anaerobic threshold in both cases. In addition to the cognitive stressors, the crane rescue maneuver contains physical stressors, since militaries require great physical ability and manual agility to manipulate the injured and lift them up with the crane. The rate of perceived exertion was also significantly higher in the crane rescue than in the low-altitude flight, which is related with the exhaustion and the physical fatigue that appear after a highly demanding task [1,5,9]. In addition, higher states of anxiety were found after the crane rescue than in the low-altitude flight, showing how the higher uncontrollability of the crane maneuvers elicited a higher anxiogenic response, since militaries have to be ready not only for the flights themselves, but also for the possible problems and difficulties while using the crane and rescuing wounded. Furthermore, the HR and SatO2 increased after the crane rescue maneuver, showing the organic adaptive response to the cardiorespiratory demands of the maneuver. This response was contrary to the low-altitude flight and other military combat situations where SatO2 decreased in the interventions [3,5,7,8,9,10,11,13,27]. The higher demands of the crane rescue maneuver could be responsible for the decreases in the horizontal jump, FEV1, PEF, and ST°, which are signals of fatigue [5].

Regarding the analysis of the participants groups, we found a significantly higher increase in SSP in the military participants compared to the control participants. This increase in stress perception does not have a negative effect on cortical arousal, memory, or strength manifestations [9]. The rate of perceived exertion of the military participants was also higher than that of the control participants, probably due to the physical demands of their different roles during the flights, in line with the decrease in the FEV1, PEF, and urine proteins [1,5,7]; as the control did not perform any activity during the flights, the perceived exertion was lower. On the other hand, we found a significant decrease in the HR of the military participants in flights due to the experience, special training, and habituation to the flight stress, as other studies have previously shown [1,6,9,28,29]. We also found a significant decrease in cortical arousal in the control participants, but not in the military participants. The new and stressful context of real helicopter flight over-stimulated the control participants, producing this fatigue of the central nervous system and decreasing cortical arousal, as described in previous research analyzing maintained combat [5,8]. This response was also similar to the results found in novel parachute jumpers, for urban combat, and in pilots with no experience, where the increase of sympathetic nervous systems caused by combat stress produced an increased organic response, increasing the metabolic, muscular, and cardiovascular response and decreasing the cortical arousal [1,8,9,15,28]. The differences between the military and control subjects suggest that military personnel suffer more stress due to the complexity of the task with low impact on cognitive arousal in comparison with control subjects, who do not perceive the same stress or demands, but suffer from cognitive impairment [29,30,31]. The previous experience, knowledge, and training seem to be protective factors that should be taken into account in future research lines.

The analysis of the HRV showed an increase in the sympathetic modulation of both groups, measured in the increase in the mean HR and Max HR, reaching peak HR values during the crane rescue maneuver, specifically in the moment where the rescuer was performing the rescue from the helicopter using the crane. The large demand of the intervention, the lack of control, and the uncertainty produce the increase in the stress response through the increase in sympathetic modulation, affecting performance, reducing cardiopulmonary capacity and strength, and momentarily stopping the urine and intestinal processes in order to prioritize organic resources to maintain survival [1,3,7,10,11,14,29]. Specifically in the low-altitude flight, we found an increased anxiety response, probably related with the danger of the maneuver, since it was conducted at a low altitude, and any minimal failure could finish in a mortal accident; nevertheless, during the flight, the sympathetic modulation decreased, showing the habituation expected from highly trained subjects [16]. This response is in line with results obtained in armies and militaries in different combat maneuvers (asymmetrical, symmetrical, urban combat, etc.), highlighting the importance of HRV control and the application of correct training to improve the autonomic response in these high-stress situations [27].

## 5. Limitations and Future Research Lines

The principal limitation of the present research was the lack of control of stress hormone, such as cortisol and alpha-amylase, as a direct measurement of physiological stress response. Another limitation to consider is the small sample of subjects, both military and civil. In military interventions like this, it is very difficult to achieve a high number of participants for various reasons: Firstly, the helicopters do not have enough space to accommodate many people, and it is quite complex to have several helicopters at the same time. Secondly, there are problems with having qualified military personnel (such as pilots) for a long time and with the number of control subjects allowed to enter the hangar. Regarding the sample size, there is another relevant limitation, which is the randomized and asymmetric distribution and division of the participants; due to the strict procedures for accessing the hangar and the complexity of schedules for both the military and control groups, the distribution was very uneven. This could possibly affect the interpretation of the results. Another limitation found was the presence of few women in the sample, probably due the general lack of women in the military population, which could affect the interpretation of the results, reducing our knowledge of how stress works on the human body with respect to gender differences. Future research might seek to address these issues.

## 6. Practical Applications

The present research showed how the use of personal, specific, durable, and wearable sensors to analyze the psychophysiological responses of helicopter crews could help to know the physiological and psychological demands of real helicopter maneuvers. This information leads us to a better understanding of the physiological and psychological stress responses of helicopter crews. The application of this information could contribute to improvement of specific training designs for both civil and military helicopter crews, but could also contribute to social wellbeing. As long as we deeply investigate, thanks to biosensors, the psychophysiological mechanisms, parameters, and biomarkers related to stress response and its mechanisms of action, we can increase the promotion and prevention of mental and physical health and reduce the impact of mental pathologies, such as PTSD and its comorbidity with other syndromes, such as depression, anxiety, insomnia, anger management, and abuse of alcohol and other substances that not only affect the person themselves, but also their relatives, friends, and coworkers.

## 7. Conclusions

The use of biosensors showed how crane rescue and low-altitude helicopter maneuvers produced an anticipatory anxiety response, showing an increased sympathetic autonomic modulation prior to the maneuvers, which were maintained during the maneuvers in both experienced and non-experienced participants. The crane rescue maneuver produced a higher maximal heart rate and decreased pulmonary capacity and strength than the low-altitude maneuver. The psychophysiological stress response was higher in the experienced than in the non-experienced participants, but both presented an anticipatory stress response before the maneuver.

## Figures and Tables

**Table 1:** Summary of participants’ characteristics.

Participants	Age	Height (cm)	Weight (kg)	Years of Service	Months in Mission	Flying Hours
Military (19)	34.52 ± 5.87	177.15 ± 6.59	77.47 ± 10.17	11.03 ± 9.13	4.48 ± 4.90	598.38 ± 638.18
Control (8)	32.37 ± 6.18	171.13 ± 8.40	65.13 ± 14.87	0.0 ± 0.0	0.0 ± 0.0	0.0 ± 0.0

Data in parentheses: number of subjects.

**Table 2:** Psychophysiological variables in the crane rescue maneuver and the low-altitude helicopter flights.

Variables	Crane Rescue Maneuver				Low-Altitude Flight			
	PRE	POST	*p*	ES	PRE	POST	*p*	ES
SSP	10 (0–30)	40 (20–60) [0.027]	0.003	1.46	0 (0–10)	10 (0–30)	0.043	1.92
RPE	6 (6–6)	11 (10–13) [0.011]	0.001	4.29	6 (6–7)	8 (6–11)	0.093	1.43
S-TM	1 (1–1)	1 (1–1)	1.000	-	1 (1–1)	1 (1–1)	1.000	-
ST° (C°)	36.7 (36.5–37.1)	36.5 (36.2–36.8)	0.027	−6.10	36.6 (36.05–36.88)	36.4 (36.3–36.6)	0.798	0.03
HR (bpm)	87 (71–111) [0.047]	75 (65–95)	0.073	−0.43	72 (67–79)	64 (61–76)	0.033	−0.50
SatO2 (%)	98 (97–99) [0.002]	97 (96–97)	0.006	−0.99	97 (96–97)	97 (96–97)	0.931	0.00
Lactate (Mmol/L)	2.1 (1.7–2.8)	3 (2.4–7.7)	0.147	0.54	4.1 (2.5–5.7)	3.2 (1.5–8.6)	0.722	0.59
IHS (N)	42 (34–51)	49 (36–51)	0.087	0.20	42 (37–48)	40 (37–51)	0.858	0.00
Horizontal Jump (m)	1.85 (1.7–1.92)	1.74 (1.7–1.8)	0.030	−0.31	1.75 (1.6–2.1)	1.9 (1.65–1.94)	0.894	0.00
Flexibility (cm)	22 (19–32)	24 (20–32.5)	0.108	0.29	25 (14.5–26)	21.5 (14–28.5)	0.953	0.00
FVC (mL)	4.5 (3.59–5.31)	4.6 (3.7–5.16)	0.244	−0.17	4.25 (4.06–4.95)	4.3 (4.22–5.36)	0.075	1.15
FEV1 (mL)	4.27 (3.55–4.54)	3.68 (3.31–4.3)	0.011	−0.82	3.82 (3.39–4.73)	3.48 (2.77–4.42)	0.083	−0.95
PEF (mL)	9.3 (8.21–11.67)	8.42 (3.64–10.08)	0.026	−0.20	11.37 (9.85–13.04)	11.03 (9.05–12.91)	0.120	−0.41
Urine Color	2 (1–3)	1 (1–2)	0.158	−0.47	1.5 (1–3)	1 (1–2)	0.257	−0.40
Urine Nitrites (Mg/dL)	1 (1–1)	1 (1–1)	1.000	-	1 (1–1)	1 (1–1)	1.000	-
Urine pH (Mg/dL)	6 (6–6)	6 (6–8)	0.065	1.89	6 (6–6)	6 (5–6)	0.317	−0.26
Urine Proteins (Mg/dL)	30 (30–100)	30 (30–30)	0.033	−0.51	65 (30–100)	30 (30–30)	0.046	−0.78
Urine Glucose (Mg/dL)	0 (0–0)	0 (0–0)	0.317	−0.26	0 (0–0)	0 (0–0)	1.000	-
USG	1.337 (1.334–1.338)	1.34 (1.334–1.339)	0.888	0.00	1.34 (1.33–1.341)	1.338 (1.338–1.34)	0.293	0.39
CFFT (Hz)	32.96 (32.3–35.48)	33.16 (30.86–34.96)	0.510	−0.09	34.07 (30.92–35.55)	31.79 (30.355–35)	0.071	−0.61
CSAI-CA	5 (5–12)	7 (5–9)	0.799	−0.06	5 (5–7)	5 (5–5)	1.000	−0.10
CSAI-SA	10 (8–12)	9 (8–14)	0.876	0.08	8 (8–8)	8 (8–10)	0.593	0.22
CSAI-SC	20 (18–20)	20 (16–20)	0.322	−0.17	20 (19–20)	20 (19–20)	0.671	−0.29
STAI-S	22 (18–27)	22 (21–31)	0.036	0.30	23 (18–29)	22 (18–23)	0.068	−0.29

Data: median, in parentheses (percentile 25–percentile 75). ES: effect size. SSP: subjective stress perception. RPE: rate of perceived exertion. S-TM: short-term memory. ST°: skin temperature. HR: heart rate. SatO2: blood oxygen saturation. IHS: isometric handgrip strength. FVC: forced vital capacity. FEV1: forced expiratory volume in one second. PEF: peak expiratory flow. USG: urine specific gravity. CFFT: critical flicker fusion threshold. CSAI-CA: cognitive anxiety. CSAI-SA: somatic anxiety. CSAI-SC: self-confidence. STAI-S: state anxiety and trait anxiety questionnaire. In brackets: statistically significant differences (*p* ≤ 0.05) between low-altitude and crane rescue maneuver helicopter flights.

**Table 3:** Psychophysiological variables in military and control participants for the helicopter flights evaluated.

Variables	Military Participants				Control Participants			
	PRE	POST	*p*	ES	PRE	POST	*p*	ES
SSP	10 (2–25)	30 (17.5–60)	0.002	1.33	12 (5–26)	15 (0–50)	0.059	1.69
RPE	6 (6–6)	10.5 (9–13)	0.001	4.38	6 (6–6)	6.5 (6–10.5)	0.066	1.77
S-TM	1 (1–1)	1 (1–1)	1.000	─	1 (1–1)	1 (1–1)	1.000	─
ST° (C°)	36.8 (36.5–37.1)	36.45 (36.1–36.8)	0.092	−3.11	36.55 (36.2–36.7)	36.5 (36.35–36.6)	0.865	0.00
HR (bpm)	85.5 (72.75–98)	75.5 (62.5–91.75)	0.029	−0.49	69 (64.75–74.75)	64.5 (63.25–69.5)	0.093	−0.35
SatO2 (%)	97 (97–98)	97 (96–97)	0.061	−0.48	97 (96.25–98.5)	97 (96–97)	0.336	−0.43
Lactate (Mmol/L)	2.5 (2–3.88)	2.5 (2.05–7.93)	0.372	0.78	3.85 (1.55–5.32)	4.8 (1.95–10.225)	0.326	0.33
IHS (N)	41 (39.25–48.75)	47.5 (38.5–51)	0.106	0.16	45 (33.25–49.25)	42.5 (31.5–50.75)	0.733	0.07
Horizontal Jump (m)	1.79 (1.6–1.91)	1.72 (1.64–1.86)	0.492	−0.09	1.905 (1.7375–2.1)	1.85 (1.73–1.98)	0.161	−0.21
Flexibility (cm)	20.5 (15.25–26.25)	21.75 (18.75–25.88)	0.350	0.25	28.75 (22.125–36.125)	30.75 (22.75–36.75)	0.446	0.08
FVC (mL)	4.52 (3.99–5.11)	4.52 (4.165–5.163)	0.913	0.17	4.24 (3.26–5.01)	4.57 (3.26–5.25)	0.400	0.47
FEV1 (mL)	4.27 (3.55–4.72)	3.64 (3.045–4.303)	0.003	−1.28	3.805 (2.95–4.36)	3.59 (2.78–4.35)	0.398	−0.20
PEF (mL)	10.64 (9.04–11.67)	9.17 (6.48–11)	0.028	−0.97	11.06 (7.9–13.04)	10.37 (7.20–13.3)	0.069	−0.21
Urine Color	2 (1–3)	1 (1–2)	0.200	−0.40	1 (1–2.75)	1 (1–1)	0.180	−0.54
Urine Nitrites (Mg/dL)	1 (1–1)	1 (1–1)	1.000	─	1 (1–1)	1 (1–1)	1.000	─
Urine pH (Mg/dL)	6 (6–6)	6 (6–7)	0.272	1.16	6 (5.25–6)	6 (5–8)	0.461	0.41
Urine Proteins (Mg/dL)	100 (30–100)	30 (30–30)	0.021	−0.51	30 (30–100)	30 (30–30)	0.083	−0.72
Urine Glucose (Mg/dL)	0 (0–0)	0 (0–0)	0.317	−0.24	0 (0–0)	0 (0–0)	1.000	─
USG	1.34 (1.34–1.34)	1.338 (1.336–1.339)	0.683	0.04	1.35 (1.334–1.34)	1.337 (1.335–1.34)	0.093	0.49
CFFT (Hz)	34.14 (32.2–35.48)	3374 (31.74–35.14)	0.520	−0.16	32.71 (30.1–35.8)	30.77 (30.265–34.05)	0.028	−0.67
CSAI-CA	5 (5–8)	5 (5–8)	0.833	−0.04	5 (5–5.75)	5 (5–6.75)	0.655	−0.09
CSAI-SA	8 (8–10)	9 (8–11.25)	0.304	0.32	8 (8–13.75)	8 (8–12.5)	0.102	−0.15
CSAI-SC	20 (18.75–20)	20 (16.75–20)	0.208	−0.36	19.5 (17.25–20)	20 (17.5–20)	1.000	0.00
STAI-S	22 (18–27.25)	21.5 (19.5–27.75)	0.127	0.25	22.5 (19–38)	23 (18.75–31)	0.465	−0.12

Data: median, in parentheses (percentile 25–percentile 75). ES: effect size. SSP: subjective stress perception. RPE: rate of perceived exertion. S-TM: short-term memory. ST°: skin temperature. HR: heart rate. SatO2: blood oxygen saturation. IHS: isometric handgrip strength. FVC: forced vital capacity. FEV1: forced expiratory volume in one second. PEF: peak expiratory flow. USG: urine specific gravity. CFFT: critical flicker fusion threshold. CSAI-CA: cognitive anxiety. CSAI-SA: somatic anxiety. CSAI-SC: self-confidence. STAI-S: state anxiety and trait anxiety questionnaire.

**Table 4:** Results of the heart rate variability variables before, during, and after the crane rescue maneuver and low-altitude helicopter flights.

Variables	Crane Rescue Maneuver				MC	Low-Altitude Flight				MC
	PRE (1)	DURING (2)	POST (3)	*p*		PRE (1)	DURING (2)	POST (3)	*p*	
Mean HR (bpm)	86.31 (78.38–90.92) [0.038]	88.31 (80.5–100.4) [0.001]	90.6 (76.6–102.43) [0.018]	0.395	─	78.61 (69.84–85.14)	68.62 (62.14–82.25)	71.81 (66.58–87.04))	0.020	1 > 2; 3 > 2
Min HR (bpm)	62.68 (59.98–68.66)	60.35 (57.3–70.82)	58.84 (55.5–69.9)	0.526	─	57.93 (56.36–64.14)	57.79 (47.92–62.85)	55.92 (52.53–66.89)	0.266	─
Max HR (bpm)	124.91 (114.03–147.84)	151.35 (121.28–215.45) [0.005]	166.93 (123.63–203.25)	0.168	─	122.7 (108.45–133.74)	109.4 (104.18–113.44)	121.04 (104.07–192.42)	0.307	─
RMSSD (ms)	28.75 ± 12.23	34.80 (16.39–38.12)	41.02 (22.5–47.3)	0.062	─	27.03 (18.63–40.37)	31.84 (21.91–40.02)	27.81 (19.67–41.73)	0.086	─
pNN50 (%)	9.74 (1.01–13.44)	11.51 (1.74–13.17)	17.83 (4.73–21.2)	0.135	─	10.37 (2.3–26.83)	9.63 (2.77–23.89)	7.34 (1.54–18.48)	0.336	─
LF (n.u)	79.65 (71.30–86.15)	76.27 (73.21–86.29)	80.73 (74.52–85.0)	0.607	─	82.71 (79.75–88.54)	79.72 (75.75–87.01)	78.38 (70.55–85.11)	0.006	1 > 2; 1 > 3; 2 > 3
HF (n.u)	2.20 (13.83–28.57)	23.70 (13.68–26.73)	19.24 (14.96–25.44)	0.607	─	17.23 (11.44–20.156)	20.23 (12.96–24.17)	21.57 (14.86–29.32)	0.006	2 > 1; 3 > 1; 3 > 2
LF/HF Ratio	3.93 (2.50–6.27)	3.22 (2.74–6.37)	4.20 (2.96–5.69)	0.607	─	4.8 (3.96–7.74)	3.94 (3.4–6.71)	3.63 (2.41–5.73)	0.006	2 < 1; 1 > 3; 2 > 3
SD1 (ms)	21.83 (10.75–25.1)	2.61 (11.59–26.96)	24.34 (9.89–33.42)	0.526	─	19.12 (13.18–28.55)	22.51 (15.49–28.30)	19.67 (13.91–29.51)	0.086	─
SD2 (ms)	56.81 (48.90–66.05)	59.6 (35.34–69.03)	67.12 (53.11–82.86)	0.062	─	52.83 (40.18–74.04)	54.34 (40.185–71.181)	53.74 (40.54–69.24)	0.000	─

Data: median, in parenthesis (percentile 25–percentile 75). MC: moment comparison. HR: heart rate. RMSSD: square root of the mean of standard deviation (SD). pNN50: differences between normal adjacent R-R electrocardiogram waves intervals > 50 ms. LF: low frequency. HF: high frequency. SD1 and SD2: standard deviations of the scattergrams 1 and 2, respectively. n.u: normalized unit. In brackets: statistically significant differences (*p* ≤ 0.05) in low-altitude and crane rescue maneuver helicopter flights.

## References

[1] Sánchez-Molina J., Robles-Pérez J.J., Clemente-Suárez V.J. (2019). Psychophysiological and fine motor skill differences of elite and non-elite soldiers in an urban combat simulation. Mil. Psychol..

[2] Tornero-Aguilera J.F., Pelarigo J.G., Clemente-Suárez V.J. (2019). Psychophysiological intervention to improve preparedness in military special operations forces. Aerosp. Med. Hum. Perform..

[3] Martin K., Périard J., Rattray B., Pyne D.B. (2020). Physiological factors which influence cognitive performance in military personnel. Hum. Factors.

[4] Curiel-Regueros A., Fernández-Lucas J., Clemente-Suárez V.J. (2019). Effectiveness of an applied high intensity interval training as a specific operative training. Physiol. Behav..

[5] Sánchez-Molina J., Robles-Pérez J.J., Clemente-Suárez V.J. (2018). Assessment of psychophysiological response and specific fine motor skills in combat units. J. Med. Syst..

[6] Vicente-Rodríguez M., Fuentes-Garcia J.P., Clemente-Suárez V.J. (2020). Psychophysiological Stress Response in an Underwater Evacuation Training. Int. J. Environ. Res. Public Health.

[7] Sánchez-Molina J., Robles-Pérez J.J., Clemente-Suárez V.J. (2017). Effect of parachute jump in the psychophysiological response of soldiers in urban combat. J. Med. Syst..

[8] Clemente-Suárez V.J., de la Vega R., Robles-Pérez J.J., Lautenschlaeger M., Fernández-Lucas J. (2016). Experience modulates the psychophysiological response of airborne warfighters during a tactical combat parachute jump. Int. J. Psychophysiol..

[9] Van den Oord Marieke H.A., Sluiter J.K., Frings-Dresen M.H. (2014). Differences in physical workload between military helicopter pilots and cabin crew. Int. Arch. Occup. Environ. Health.

[10] Human Factors and Ergonomics Society (2002). Human factors in aircraft accidents: A holistic approach to intervention strategies. Proceedings of the Human Factors and Ergonomics Society Annual Meeting.

[11] Taverniers J., Smeets T., Bue S.L., Syroit J., Van Ruysseveldt J., Pattyn N., von Grumbkow J. (2011). Visuo-spatial path learning, stress, and cortisol secretion following military cadets’ first parachute jump: The effect of increasing task complexity. Cogn. Affect. Behav. Neurosci..

[12] Gibb R., Ercoline B., Scharff L. (2011). Spatial disorientation: Decades of pilot fatalities. Aviat. Space Environ. Med..

[13] Debevec T., Amon M., Keramidas M.E., Kounalakis S.N., Pišot R., Mekjavic I.B. (2010). Normoxic and hypoxic performance following 4 weeks of normobaric hypoxic training. Aviat. Space Environ. Med..

[14] Vicente-Rodriguez M., Clemente-Suárez V. (2020). Psychophysiological anxiety response of a rescue helicopter crew in a crane rescue manoeuvre. BMJ Mil. Health.

[15] Hormeño-Holgado A.J., Clemente-Suárez V.J. (2019). Effect of different combat jet manoeuvres in the psychophysiological response of professional pilots. Physiol. Behav..

[16] Tornero Aguilera J.F., Gil-Cabrera J., Clemente-Suarez V.J. (2020). Determining the psychophysiological responses of military aircrew when exposed to acute disorientation stimuli. BMJ Mil. Health.

[17] Hormeño-Holgado A.J., Perez-Martinez M.A., Clemente-Suárez V.J. (2019). Psychophysiological response of air mobile protection teams in an air accident manoeuvre. Physiol. Behav..

[18] Sánchez-Molina J., Robles-Pérez J.J., Clemente-Suárez V.J. (2017). Respuesta fisiológica de una unidad paracaidista en combate urbano. Arch. Med. Deporte.

[19] Peng H.T., Savage E., Vartanian O., Smith S., Rhind S.G., Tenn C., Bjamason S. (2016). Performance evaluation of a salivary amylase biosensor for stress assessment in military field research. J. Clin. Lab. Anal..

[20] Kim J., Valdés-Ramírez G., Bandodkar A.J., Jia W., Martinez A.G., Ramírez J., Mercier P., Wang J. (2014). Non-invasive mouthguard biosensor for continuous salivary monitoring of metabolites. Analyst.

[21] Borg G.A., Noble B.J. (1974). Perceived exertion. Exerc. Sport Sci. Rev..

[22] Hormeño-Holgado A.J., Clemente-Suárez V.J. (2019). Psychophysiological Monitorization in a Special Operation Selection Course. J. Med. Syst..

[23] Clemente-Suárez V., González-Ravé J., Navarro-Valdivielso F. (2014). Short-term periodized aerobic training does not attenuate strength capacity or jump performance in recreational endurance athletes. Acta Physiol. Hung..

[24] Armstrong L.E., Herrera Soto J.A., Hacker Jr F.T., Casa D.J., Kavouras S.A., Maresh C.M. (1998). Urinary indices during dehydration, exercise, and rehydration. Int. J. Sport Nutr..

[25] Massaro S., Pecchia L. (2019). Heart rate variability (HRV) analysis: A methodology for organizational neuroscience. Organ. Res. Methods.

[26] Caminal P., Sola F., Gomis P., Guasch E., Perera A., Soriano N., Mont L. (2018). Validity of the Polar V800 monitor for measuring heart rate variability in mountain running route conditions. Eur. J. Appl. Physiol..

[27] Bustamante-Sánchez Á., Tornero-Aguilera J.F., Fernández-Elías V.E., Hormeño-Holgado A.J., Dalamitros A.A., Clemente-Suárez V.J. (2020). Effect of Stress on Autonomic and Cardiovascular Systems in Military Population: A Systematic Review. Cardiol. Res. Pract..

[28] Tornero-Aguilera J.F., Robles-Pérez J.J., Clemente-Suárez V.J. (2018). Use of psychophysiological portable devices to analyse stress response in different experienced soldiers. J. Med. Syst..

[29] Delgado-Moreno R., Robles-Pérez J.J., Aznar-Laín S., Clemente-Suárez V.J. (2019). Effect of Experience and Psychophysiological Modification by Combat Stress in Soldier’s Memory. J. Med. Syst..

[30] Tornero-Aguilera J.F., Robles-Pérez J.J., Clemente-Suárez V.J. (2017). Effect of combat stress in the psychophysiological response of elite and non-elite soldiers. J. Med. Syst..

[31] Ivie D., Garland B. (2011). Stress and burnout in policing: Does military experience matter?. Polic. Int. J. Police Strateg. Manag..

